# Clinical characteristics and outcomes of B-ALL with *ZNF384* rearrangements: a retrospective analysis by the Ponte di Legno Childhood ALL Working Group

**DOI:** 10.1038/s41375-021-01199-0

**Published:** 2021-03-10

**Authors:** Shinsuke Hirabayashi, Ellie R. Butler, Kentaro Ohki, Nobutaka Kiyokawa, Anke K. Bergmann, Anja Möricke, Judith M. Boer, Hélène Cavé, Giovanni Cazzaniga, Allen Eng Juh Yeoh, Masashi Sanada, Toshihiko Imamura, Hiroto Inaba, Charles Mullighan, Mignon L. Loh, Ulrika Norén-Nyström, Agata Pastorczak, Lee-Yung Shih, Marketa Zaliova, Ching-Hon Pui, Oskar A. Haas, Christine J. Harrison, Anthony V. Moorman, Atsushi Manabe

**Affiliations:** 1 Department of Pediatrics, Hokkaido University Graduate School of Medicine, Sapporo, Japan; 2 Leukaemia Research Cytogenetics Group, Wolfson Childhood Cancer Research Centre, Translational and Clinical Research Institute, Newcastle University, Newcastle upon Tyne, UK; 3 Department of Pediatric Hematology and Oncology Research, National Research Institute for Child Health and Development, Tokyo, Japan; 4 Hannover Medical School, Institute of Human Genetics, Hannover, Germany; 5 Department of Pediatrics, Christian-Albrechts-University Kiel and University Medical Center Schleswig-Holstein, Kiel, Germany; 6 Princess Máxima Center for Pediatric Oncology, Utrecht, Netherlands; 7 Oncode Institute, Utrecht, Netherlands; 8 Department of Genetics, Robert Debré Hospital and University of Paris, Paris, France; 9 Centro Ricerca Tettamanti, Pediatric Clinic University of Milano-Bicocca, Monza, Italy; 10 Khoo Teck Puat - National University Children’s Medical Institute, Yong Loo Lin School of Medicine, National University of Singapore, Singapore, Singapore; 11 Department of Advanced Diagnosis, Clinical Research Center, National Hospital Organization Nagoya Medical Center, Nagoya, Japan; 12 Department of Pediatrics, Graduate School of Medical Science, Kyoto Prefectural University of Medicine, Kyoto, Japan; 13 St Jude Children’s Research Hospital and Department of Pediatrics, College of Medicine, The University of Tennessee Health Science Center, Memphis, TN USA; 14 Department of Pediatrics, Benioff Children’s Hospital and the Helen Diller Family Comprehensive Cancer Center, University of California, San Francisco, San Francisco, CA USA; 15 Department of Clinical Sciences, Pediatrics, Umeå University, Umeå, Sweden; 16 Department of Pediatric, Oncology, Hematology and Diabetology, Medical University of Łódź, Poland, CA Poland; 17 Division of Hematology-Oncology, Chang Gung Memorial Hospital at Linkou and Chang Gung University, Taoyuan, Taiwan; 18 CLIP, Department of Paediatric Haematology/Oncology, Charles University Prague, 2nd Faculty of Medicine, Prague, Czech Republic; 19 Children’s Cancer Research Institute, Vienna, Austria

**Keywords:** Acute lymphocytic leukaemia, Cancer genetics

## To the Editor

B-acute lymphoblastic leukemia (B-ALL) comprises a wide variety of subtypes with diverse clinical and biological features and outcomes. Risk-stratified and targeted therapy according to genetic subtype has improved B-ALL outcomes [1]. Next generation sequencing (NGS) has identified several novel subtypes, including one with *ZNF384* rearrangements involving *ZNF384* located at position 12p13.31 [2–7]. Interestingly, patients with this subtype appear to express various leukemic phenotypes, including B-ALL (with or without aberrant expression of myeloid markers) and B/myeloid mixed phenotype acute leukemia (MPAL). In this regard, ~5% of childhood B-ALL, ~10% of adult B-ALL, and 48% of B/Myeloid MPAL cases have been observed to harbor *ZNF384* rearrangements [2–4, 8]. RNA sequencing and conventional methods have identified more than 10 fusion partners of *ZNF384* rearrangements [2–7], but the clinical significance of each fusion partner remains unclear due to the small number of reported cases. Here we describe the clinical characteristics and outcomes of the largest series of B-ALL cases with *ZNF384* rearrangements reported to date.

We studied a total of 218 cases with *ZNF384* rearrangements identified by 16 international consortia (Supplementary Table 1) belonging to the Ponte di Legno Childhood ALL Working group. Patients were diagnosed with B-ALL between 1992 and 2018 using standard morphological and immunophenotypic criteria. The focus of this study was the clinical relevance of *ZNF384* fusions in B-ALL. We excluded MPAL cases because they are frequently treated off-study and can receive hybrid therapies [9]. *ZNF384* rearrangements were detected by fluorescence in situ hybridization (FISH), reverse transcription polymerase chain reaction, and/or NGS as per individual study group criteria. Three groups identified 18 cases with *ZNF384* rearrangements among patients with relapsed or refractory ALL, and these cases were excluded from the outcome analysis. We collated data on the clinical and biological characteristics of patients with *ZNF384*-rearrangements from each study group and analyzed their association with clinical outcomes.

Overall survival (OS) was calculated from the time from diagnosis to death, while event-free survival (EFS) was defined as the time from diagnosis to induction failure, relapse, a second tumor, or death; time was censored at the date of last patient contact if no event occurred. Relapse rate was defined as the time from diagnosis to relapse censoring at other events. The Kaplan–Meier method was used to estimate survival rates, and evaluation of the equality of the survivorship functions in different subgroups was performed using the two-sided log-rank test. Univariate Cox regression models were used to determine hazard ratios (HR). Other comparisons were performed using the $\chi ^2$ test or Fisher’s exact test as appropriate. All probability (*P*) values were two-sided, and *P* values < 0.05 were considered statistically significant. All analyses were performed using Intercooled Stata (Statacorp 2015 Stata Statistical Software Release 14; StataCorp, College Station, TX).

Among the 218 patients with B-ALL and *ZNF384* rearrangements, information on the partner gene was available for 193 cases with frequencies of 43% for *EP300*, located at position 22q13.2 (*n* = 83, 31% for *TCF3* at 19p13.3 (*n* = 60), 9% for *TAF15* at 17q12 (*n* = 17), 8% for *CREBBP* at 16p13.3 (*n* = 15), and 9% for others (*n* = 18) (Supplementary Table 2). The 18 other partner genes included six cases of *EWSR1* and one case of each of *ARID1B, BMP2K, CLLORF74, CCAR1, CLTC, DUX4, NIPBL, SEC24B, SMARCA2, USP25*. In 2 cases the partner gene could not be determined but involvement of *EP300, TCF3, TAF15, and CREBBP* was excluded. Data on demographics are shown in Table 1. The female to male ratio was 1:1. The age distribution differed according to partner gene: Patients with *EP300-ZNF384* were older (median age, 11 years), while patients with *TCF3-ZNF384* were younger (median age, 5 years) (*P* < 0.001). There were no statistical differences in the distribution of data on National Cancer Institute risk group, ethnicity, leukocyte count, or CNS status according to the partner gene involved (Table 1). Immunophenotypically, the majority of the cases expressed myeloid-associated antigens CD13 (38–100%) and CD33 (78–100%), and a relatively large number of cases were negative for CD10 (14–51%) (Supplementary Table 3), as reported previously [3, 4]. Complete hematological remission was achieved in 99% of the cases. In total, 31% of patients were treated as “high risk” according to protocol, and 23% of the patients received a stem cell transplant in the first remission. Minimal residual disease (MRD) data were available for 77 patients; for 18 (23%) of these, MRD was positive (median, 2.54%; range, 0.14–25.6%) at the end of induction.

**Table 1:** Demographic features of patients with B-ALL and *ZNF384* rearrangements stratified by partner genes.

	Total *n* (%)	*EP300 n (%)*	*TCF3 n (%)*	*TAF15 n (%)*	*CREBBP n (%)*	Other^a^ *n* (%)	Missing^b^ *n* (%)	*p* value
Total, n (%)	218 (100)	83	60	17	15	18	25	
Sex, *n*(%)								
Male	104 (50)	36 (47)	28 (47)	11 (65)	9 (64)	11 (65)	9 (36)	0.381
Female	104 (50)	40 (53)	31 (53)	6 (35)	5 (36)	6 (35)	16 (64)	
Unknown/Missing	10	7	1	0	1	1	0	
Age (years)								
Median	9.00	11.00	5.00	8.00	6.00	7.00	12.00	
1–9	115 (55)	32 (42)	44 (75)	11 (65)	9 (64)	10 (59)	9 (36)	0.001
10–14	65 (31)	28 (37)	14 (24)	5 (29)	2 (14)	4 (24)	12 (48)	
15–18	24 (12)	15 (20)	0 (0)	1 (6)	3 (21)	1 (6)	4 (16)	
19–25	4 (2)	1 (1)	1 (2)	0 (0)	0 (0)	2 (12)	0 (0)	
Unknown/Missing	10	7	1	0	1	1	0	
Year of diagnosis								
1992–2007	90 (43)	26 (34)	26 (44)	8 (47)	5 (36)	2 (13)	23 (92)	0.171
2008–2018	117 (57)	50 (66)	33 (56)	9 (53)	9 (64)	14 (88)	2 (8)	
Unknown/Missing	11	7	1	0	1	2	0	
Race								
Asian	62 (48)	20 (44)	28 (72)	5 (45)	4 (40)	2 (22)	3 (20)	0.083
White	59 (46)	23 (51)	11 (28)	6 (55)	5 (50)	6 (67)	8 (53)	
Other	8 (6)	2 (4)	0 (0)	0 (0)	1 (10)	1 (11)	4 (27)	
Unknown/Missing	89	38	21	6	5	9	10	
WBC count (10^6^/L)								
<50,000	151 (74)	59 (79)	37 (64)	13 (81)	8 (57)	15 (88)	19 (76)	0.088
>50,000	54 (26)	16 (21)	21 (36)	3 (19)	6 (43)	2 (12)	6 (24)	
Unknown/Missing	13	8	2	1	1	1	0	
NCI risk group								
Standard Risk	73 (35)	24 (32)	24 (41)	8 (50)	5 (36)	8 (47)	4 (16)	0.524
High Risk	133 (65)	52 (68)	34 (59)	8 (50)	9 (64)	9 (53)	21 (84)	
Missing	12	7	2	1	1	1	0	
CNS3 disease at diagnosis								
Yes	5 (3)	1 (2)	1 (2)	0 (0)	1 (8)	1 (7)	1 (4)	0.567
No	165 (97)	56 (98)	45 (98)	15 (100)	11 (92)	14 (93)	24 (96)	
Unknown/Missing	48	26	14	2	3	3	0	
Traumatic lumbar puncture								
Yes, Blasts	4 (4)	0 (0)	2 (6)	0 (0)	1 (14)	0 (0)	1 (20)	0.386
Yes, No Blasts	6 (5)	4 (9)	2 (6)	0 (0)	0 (0)	0 (0)	0 (0)	
No	99 (91)	42 (91)	29 (88)	9 (100)	6 (86)	9 (100)	4 (80)	
Unknown/Missing	109	37	27	8	8	9	20	
Immunophenotype								
B-Lineage	205 (100)	75 (99)	57 (97)	17 (100)	14 (93)	17 (100)	25 (100)	N/A
T-Lineage	0 (0)	0 (0)	0 (0)	0 (0)	0 (0)	0 (0)	0 (0)	
Unknown/Missing	13	8	3	0	1	1	0	
BM Blasts at diagnosis								
<20	20 (34)	6 (33)	3 (21)	2 (100)	2 (67)	1 (50)	6 (32)	0.161
20–39	13 (22)	4 (22)	6 (43)	0 (0)	0 (0)	0 (0)	3 (16)	
40–59	11 (19)	6 (33)	0 (0)	0 (0)	0 (0)	0 (0)	5 (26)	
60–80	7 (12)	1 (6)	3 (21)	0 (0)	0 (0)	1 (50)	2 (11)	
>80	7 (12)	1 (6)	2 (14)	0 (0)	1 (33)	0 (0)	3 (16)	
Unknown/Missing	160	65	46	15	12	16	6	
Minimal residual disease (Neg < 0.01%)								
Positive	18 (23)	5 (16)	6 (32)	0 (0)	1 (17)	4 (33)	2 (33)	0.491
Negative	59 (77)	26 (84)	13 (68)	3 (100)	5 (83)	8 (67)	4 (67)	
Unknown/Missing	141	52	41	14	9	6	19	
Complete remission achieved								
Yes	199 (99)	74 (100)	56 (98)	13 (100)	14 (100)	17 (100)	25 (100)	0.721
No	1 (1)	0 (0)	1 (2)	0 (0)	0 (0)	0 (0)	0 (0)	
Unknown/Missing	18	9	3	4	1	1	0	
Stem cell transplant received								
Yes	42 (23)	20 (26)	13 (25)	2 (13)	4 (29)	3 (20)	0 (0)	0.85
No	138 (77)	58 (74)	39 (75)	13 (87)	10 (71)	12 (80)	6 (100)	
Unknown/Missing	38	5	8	2	1	3	19	
Treatment risk groups								
Non-high risk	144 (69)	58 (73)	31 (54)	13 (76)	9 (64)	11 (65)	22 (96)	0.215
High risk	64 (31)	22 (28)	26 (46)	4 (24)	5 (36)	6 (35)	1 (4)	
Unknown/Missing	10	3	3	0	1	1	2	

^a^The other group includes 6 cases of *EWSR1* and 1 case each of *ARID1B*, *BMP2K*, *CLLORF74*, *CCAR1*, *CLTC*, *DUX4*, *NIPBL*, *SEC24B*, *SMARCA2*, *USP25*; plus two cases where testing showed that the partner gene was not one of the four common genes.

^b^Missing group includes cases where information about the partner gene was not provided or where the involvement of *ZNF384* was confirmed by FISH only.

**Fig. 1:**
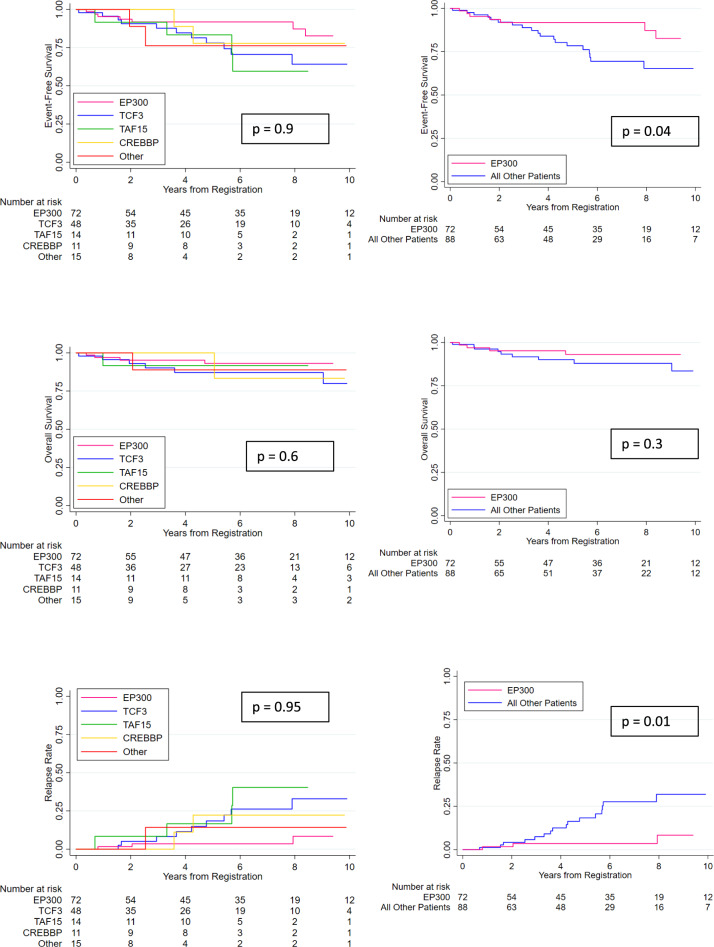
Outcomes of patients with *ZNF384* rearrangement-related ALL. Outcomes of patients with *ZNF384* rearrangement-related ALL according to partner gene and *EP300-ZNF384* ALL compared with all other patients. Of note, outcome data were missing for 15 patients, and further 18 and 25 patients were excluded for selection bias and missing partner gene information, respectively.

After a median follow-up of 5.8 years, the 5-year EFS rate was 85% (95% confidence interval [CI], 78–90%), and the 5-year OS rate was 91% (95% CI, 85–95%) for all patients. There was no difference in survival rate by treatment period, by country, or by region of origin (data not shown). Data on the outcome of patients with *ZNF384*-rearranged ALL according to partner gene are summarized in Fig. 1. Univariate and multivariate analyses revealed outcome heterogeneity by partner gene (Supplementary Table 4). Patients with an *EP300-ZNF384* fusion had a significantly lower cumulative relapse rate at 5 years compared with the remaining patients, 4 % (95% CI, 1–13%) vs. 18% (11–31 %) (HR, 0.20, [95%CI, 0.06 –0.67], *P* = 0.01). The corresponding EFS was 92 % (95% CI, 81–97 %) vs. 78 % (95% CI, 66–87%) (*P* = 0.037), and OS was 93 % (95% CI, 82–97 %) *vs*. 90% (95% CI, 80–95%) (*P* = 0.289), suggesting that relapses of other *ZNF384* rearrangements were salvageable. Multivariate analysis, adjusting for sex, age, WBC, and treatment period did not alter the results (supplementary Table 4).

Additional genetic abnormalities were detected by multiplex ligation-dependent probe amplification (*n* = 91), single nucleotide polymorphism array analysis (*n* = 63), RNA sequencing (*n* = 117), whole-exome sequencing (*n* = 18), and whole-genome sequencing (*n* = 12) performed by each international consortia (Supplementary Table 5). Commonly deleted genes included those frequently recurrent in ALL [10], such as *ETV6* (*n* = 35, 24 %), *CDKN2A/2B* (*n* = 23, 16%), and *IKZF1* (*n* = 20, 14%); and those frequently mutated within the RAS pathway, such as *FLT3* (*n* = 10, 14%). The distribution of deletions did not differ significantly between fusion partners (Supplementary Table 6, Supplementary Fig. 1). There were no significant associations between genomic deletions and prognosis for any of the fusion partners investigated (Supplementary Table 7). Deletions of *CDKN2A*/*CDKN2B*, which were found in 23 (16%) of the cases, tended to be associated with a higher mortality risk (HR, 3.86 [95%CI, 0.96–15.48], *P* = 0.057).

A recent study of monozygotic twins revealed that *TCF3-ZNF384* can occur *in utero*, suggesting that the *TCF3-ZNF384* fusion gene is crucial to initiate ALL [11]. The chromosomal translocation from which the chimeric fusion gene arises may have formed a pre-leukemic clone, which acquired additional mutations to fully transform into overt leukemia several years after birth, as has been demonstrated for *ETV6–RUNX1* ALL [12]. In contrast, ALL with *EP300-ZNF384* was present in patients older than those with other *ZNF384*-related rearrangements. The biological background for each fusion partner warrants further investigation.

In this study, 28 % of children with *EP300-ZNF384* were allocated to the high-risk group because of older age, while 46% of patients with *TCF3-ZNF384* were allocated to the high-risk group because of high leukocyte counts and poor initial responses. The patients with *EP300-ZNF384* ALL had a lower cumulative relapse rate than the remaining patients particularly those patients with *TCF3-ZNF384*. However, each group of patients might benefit from MRD-directed treatment in order to avoid over- and under-treatment as well as targeted therapy. In this regard, immunotherapy, such as anti-CD19 chimeric antigen receptor T-cell therapy (CAR-T therapy), should be used carefully, if at all, in patients with *ZNF384* rearrangements because of the risk of lineage switch to myeloid leukemia as a cause of relapse [13]. In fact, lineage switch from ALL to acute myeloid leukemia after CAR-T therapy has been observed in one patient with *TCF3-ZNF384* [14]. In addition, relapse tends to occur late, so that the EFS curve does not plateau at 5 years (Fig. 1). Indeed, we observed two patients with *TCF3-ZNF384*-positive ALL relapsing more than 10 years after diagnosis [15]. Taken together, additional studies are needed to develop an optimal treatment strategy for those with poor initial responses, although allogeneic stem cell transplantation is unlikely to be indicated.

*ZNF384* fusions are an enigmatic group of gene fusions which span the ALL-MPAL disease spectrum and are not readily detected and characterized by traditional genetic testing [8]. It is definitey required to screen by FISH, reverse transcription polymerase chain reaction or RNA sequencing. The major strengthen of this study is that it has collated a large well-annoatated cohort of *ZNF384*-fusion patients which while not uninformly treated were all diagnosed with ALL and treated as such. As with all retrospective consortia-based studies the limitations are heterogeneity in terms of recrtuiment period and treatment decisions and pathways. Nonetheless, we provide good evidence that among B-ALL patients with a *ZNF384* fusion the partner gene is associated with demographic features and influences outcome, specifically with *EP300-ZNF384* being associated with a lower risk of relapse. We opted to exlude MPAL cases because, historically, they have not been treated uniformly. A recent international cooperative study has defined a consensus treatment staregy for MPAL patients [9]. This initiative coupled with improved diagnostic genomic testing will enable future prospective studies to clarify the clinical relevance of the fusions in both ALL and MPAL.

## Supplementary information

Supplementary Figure 1

Supplementary Table 1

Supplementary Table 2

Supplementary Table 3

Supplementary Table 4

Supplementary Table 5

Supplementary Table 6

Supplementary Table 7

## References

[1] Hunger SP, Mullighan CG (2015). Acute lymphoblastic leukemia in children. N Engl J Med.

[2] Yasuda T, Tsuzuki S, Kawazu M, Hayakawa F, Kojima S, Ueno T (2016). Recurrent DUX4 fusions in B cell acute lymphoblastic leukemia of adolescents and young adults. Nat. Genet.

[3] Liu YF, Wang BY, Zhang WN, Huang JY, Li BS, Zhang M (2016). Genomic profiling of adult and pediatric B-cell acute lymphoblastic leukemia. EBioMedicine..

[4] Hirabayashi S, Ohki K, Nakabayashi K, Ichikawa H, Momozawa Y, Okamura K (2017). ZNF384-related fusion genes define a subgroup of childhood B-cell precursor acute lymphoblastic leukemia with a characteristic immunotype. Haematologica..

[5] Li JF, Dai YT, Lilljebjorn H, Shen SH, Cui BW, Bai L (2018). Transcriptional landscape of B cell precursor acute lymphoblastic leukemia based on an international study of 1,223 cases. Proc Natl Acad Sci USA.

[6] Gu Z, Churchman ML, Roberts KG, Moore I, Zhou X, Nakitandwe J (2019). PAX5-driven subtypes of B-progenitor acute lymphoblastic leukemia. Nat Genet.

[7] Zaliova M, Stuchly J, Winkowska L, Musilova A, Fiser K, Slamova M (2019). Genomic landscape of pediatric B-other acute lymphoblastic leukemia in a consecutive European cohort. Haematologica..

[8] Alexander TB, Gu Z, Iacobucci I, Dickerson K, Choi JK, Xu B (2018). The genetic basis and cell of origin of mixed phenotype acute leukaemia. Nature..

[9] Hrusak O, de Haas V, Stancikova J, Vakrmanova B, Janotova I, Mejstrikova E (2018). International cooperative study identifies treatment strategy in childhood ambiguous lineage leukemia. Blood..

[10] Steeghs EMP, Boer JM, Hoogkamer AQ, Boeree A, de Haas V, de Groot-Kruseman HA (2019). Copy number alterations in B-cell development genes, drug resistance, and clinical outcome in pediatric B-cell precursor acute lymphoblastic leukemia. Sci Rep..

[11] Bueno C, Tejedor JR, Bashford-Rogers R, Gonzalez-Silva L, Valdes-Mas R, Agraz-Doblas A (2019). Natural history and cell of origin of TC F3-ZN F384 and PTPN11 mutations in monozygotic twins with concordant BCP-ALL. Blood..

[12] Greaves M (2018). A causal mechanism for childhood acute lymphoblastic leukaemia. Nat Rev Cancer.

[13] Novakova M, Zaliova M, Fiser K, Vakrmanova B, Slamova L, Musilova A, et al. DUX4r, ZNF384r and PAX5-P80R mutated B-cell precursor acute lymphoblastic leukemia frequently undergo monocytic switch. Haematologica. 2020. 10.3324/haematol.2020.250423 PMC8327733 32646889

[14] Oberley MJ, Gaynon PS, Bhojwani D, Pulsipher MA, Gardner RA, Hiemenz MC (2018). Myeloid lineage switch following chimeric antigen receptor T-cell therapy in a patient with TCF3-ZNF384 fusion-positive B-lymphoblastic leukemia. Pediatr Blood Cancer.

[15] Nishimura A, Hasegawa D, Hirabayashi S, Kanabuchi S, Yamamoto K, Aiga S (2019). Very late relapse cases of TCF3-ZNF384-positive acute lymphoblastic leukemia. Pediatr Blood Cancer.

