# Supplementary figures and images for: Clinical characteristics and outcomes of B-ALL with ZNF384 rearrangements: a retrospective analysis by the Ponte di Legno Childhood ALL Working Group
Source: Leukemia. 2021 Mar 10;35(11):3272–7. doi: 10.1038/s41375-021-01199-0 (PMC8550960; doi:10.1038/s41375-021-01199-0)

Supplementary Figure 1.

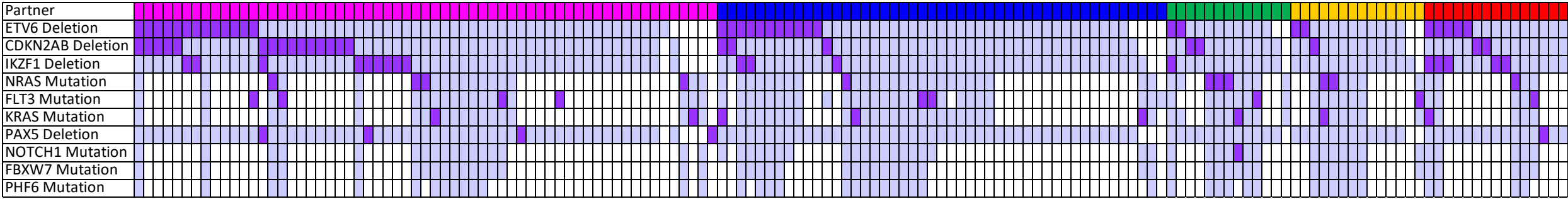

|                   |          |          |       |        |       |
|-------------------|----------|----------|-------|--------|-------|
| Partner           | EP300    | TCF3     | TAF15 | CREBBP | Other |
| Deletion/Mutation | Positive | Negative |       |        |       |

Supplement: Supplementary file 1 — Supplementary Figure 1 [file 41375_2021_1199_MOESM1_ESM.pdf]
